# Self-compassion and work engagement among Chinese nurses: exploring mediating effects of depression, anxiety, and stress

**DOI:** 10.3389/fpubh.2024.1420384

**Published:** 2024-09-23

**Authors:** Yan Yang, Hongjuan Chang, Shuangxi Guo, Xiao Lei Gao, Lina Wang, Anna Ma

**Affiliations:** ^1^School of Nursing, Xinxiang Medical University, Xinxiang, China; ^2^School of Medicine, Wuhan University of Science and Technology, Wuhan, China; ^3^The First Affiliated Hospital of Xinxiang Medical University, Xinxiang, China

**Keywords:** self-compassion, negative emotions, work engagement, nurses, structural equation modelling

## Abstract

**Objective:**

Work engagement significantly influences both the quality of nursing care and nurses’ job performance. In this study, we aimed to explore the mediating effects of negative emotions on the relationship between self-compassion and work engagement among Chinese nurses.

**Method:**

A cross-sectional study was performed on nurses in a tertiary A hospital located in Henan province from September, 2023 to December, 2023. Custom-designed digital surveys were disseminated to gather pertinent data. Structural Equation Modelling (SEM) were utilised to analyse the data and determine relationships among self-compassion, negative emotions and work engagement.

**Results:**

A total of 1,201 nurses were included. According to the statistical model, self-compassion (β = 0.116, CI: −0.036 to −0.008, *p* < 0.001) and negative emotions (β = −0.372, CI: −0.053 to −0.033, *p* < 0.001) were correlated with work engagement. Furthermore, our analysis revealed that negative emotions partially mediated the relationship between self-compassion and work engagement (β = 0.174, CI: −0.066 to −0.020, *p* < 0.01).

**Conclusion:**

These findings indicate that incorporating self-compassion and negative emotion regulation in interventions targeting work engagement may enhance the overall level of work engagement among nurses, thereby improving job satisfaction and the quality of patient care.

## Introduction

Work engagement is characterised by a positive, affective-motivational state marked by elevated energy levels, unwavering dedication and a profound focus on professional responsibilities (1). In the past two decades, there has been a notable escalation in the number of studies dedicated to the exploration of work engagement. The current landscape of work engagement research reveals several notable trends. One particularly significant development involves the exploration of engagement as a dynamic phenomenon, subject to fluctuations within individuals across various temporal and situational contexts (2). This includes investigations into daily work engagement, as well as the examination of weekly and episodic variations in an individual’s engagement levels. Furthermore, there is a growing body of research exploring the intersection between engagement and human resource management (HRM) systems and practices, with a specific focus on their impact on employee work experiences (3). Another noteworthy trend centres around the intricate relationship between leadership and engagement, as evidenced by studies such as those conducted by Prochazka et al. (4) and Decuypere and Schaufeli (5). These highlight a heightened interest in understanding how different leadership styles, behaviors and approaches influence the engagement levels of employees. Khusanova et al. (6) investigated diverse workplaces to confirm the associations among job meaningfulness, work engagement and performance. The findings indicated a positive relationship between job meaningfulness and work engagement, highlighting the interconnectedness between work engagement and performance.

Although some studies revealed the relationship between work engagement and individual places, practices and leaders, whether psychological flexibility affects work engagement remained unclear. Therefore, this study proposed to test the relationship between self-compassion and work engagement, and the role of depression, anxiety and stress (DASS) among Chinese nurses. The relationships between the variables mentioned above are described in the following sections.

Work engagement is a critical construct as it is intricately linked to the well-being of staff and the overall performance of their roles (37). Work engagement encompasses three key dimensions: vigor, dedication and absorption (38). The attribute of vigor at the workplace signifies the mental fortitude and dynamic energy that professionals bring to their daily tasks. Dedication, on the other hand, reflects an unwavering devotion to one’s job, accompanied by a deep sense of purpose, the willingness to tackle challenges, a state of inspiration, and an emotional investment of pride and eagerness in one’s work. Additionally, being engrossed in one’s work signifies a level of complete concentration and profound involvement in job-related activities. Employees who benefit from positive workplace environments, characterized by just compensation, employment continuity, and opportunities for advancement, tend to report increased job satisfaction and a stronger sense of job security. Existing studies have shown that the level of engagement among nursing staff is significantly linked to the quality of nursing care and can foresee the dynamics of nurse–patient interactions (39), as well as the intentions of nurses to leave their positions (40). A research conducted by Babenko et al. (7) revealed that physicians who practice self-compassion exhibit higher levels of work engagement and report lower levels of fatigue due to job demands, leading to greater overall satisfaction with their professional lives. Additionally, another study observed a positive association between self-compassion and work engagement among employees in the Netherlands (41). The Self-Determination Theory (SDT) and its associated psychological constructs are utilized to hypothesize the impact of self-compassion on employee engagement in the workplace, SDT categorizes motivation into extrinsic, intrinsic, and amotivation types (41). Evidence from select research indicates that intrinsic motivation plays a pivotal role in fostering a heightened level of engagement among employees.

Self-compassion, the act of extending understanding and kindness towards one’s own internal struggles, proves to be a constructive method for navigating challenging thoughts and emotions, fostering both mental and physical well-being (8). Self-compassion revolves around preserving inner balance when confronted with unfavourable situations, embracing the current moment of life as it unfolds. Self-compassion can be directed inward when one undergoes suffering that is not attributable to personal fault instances where external life circumstances are inherently painful or challenging to endure. Additionally, it serves as a vital internal asset, enabling individuals to discover optimism and inner resilience in the face of life’s challenges. The primary advantages of self-compassion are associated with the effective coping mechanisms and stress management strategies employed by individuals who possess high levels of self-compassion. Additionally, these individuals demonstrate proficiency in self-regulation, enabling them to navigate and maintain a harmonious spectrum of both positive and negative emotions (9). Drawing from the research findings, it is evident that incorporating self-compassion education serves as a highly effective approach to enhance positive emotions and mitigate negative emotions among nursing students (10).

The positive outcomes of emotional experiences can include enhanced self-efficacy and a sense of empowerment, both of which are essential for work engagement. These elements have been validated through empirical research within the frameworks of social cognitive theory and cognitive motivation theories (42). In the present study, the negative emotions included the depression, the anxiety and stress. Chen et al. (11) discovered a pronounced link between experiencing negative emotions and the practice of self-compassion. This implies that nurturing a deep sense of self-compassion can be beneficial for people facing adversity. By doing so, it can help to lower the degree of self-blame one might experience and alleviate the constant reflection on negative events, which in sum, can lead to improved mental health and well-being (12). Self-compassion plays a pivotal role in alleviating negative emotional responses within the occupational sphere (43). Conversely, emotions, being interconnected with job performance, can function as a mediator in the workplace (39). Additionally, a strong correlation seems to exist between the extent of engagement in one’s work and the ability to manage negative emotions effectively. However, the current research tends to concentrate on physicians and patients, often overlooking nurses in such investigative studies.

**Figure 1:**
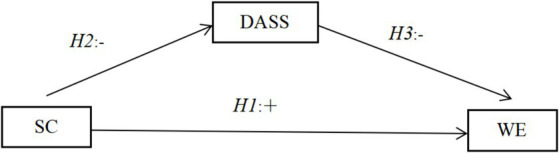
Hypothesized model of relationships among self-compassion, negative emotions and work engagement.

Hence, our study aims to elucidate the interplay among Chinese nurses’ work engagement, negative emotions and self-compassion. We hypothesised that self-compassion is positively correlated with degree of work engagement. Negative emotion plays the role of partially mediated between self-compassion and work engagement. We constructed a hypothetical model, which is depicted in Figure 1.

## Methods

### Participants and procedure

To explore the levels of work engagement, self-compassion, and negative emotions among nursing professionals, our study employed a structured assessment through an online, confidential questionnaire. The cross-sectional study, conducted in Henan province, involved a convenience sampling method applied at a tertiary A hospital.

Our research utilized a digital recruitment strategy, active between September 1st and December 1st, 2023. To qualify for the study, the participants consisted of nurses that had to meet the following criteria: (1) to be a licensed registered nurse; (2) actively working during the survey period; (3) with at least 6 months of experience in their current healthcare facility. Additionally, our research did not encompass the participation of additional healthcare specialists.

The web-based survey, facilitated by a digital platform (https://www.wjx.cn, accessed from September 1, 2023 to December 1, 2023). The Nursing Department distributed information to the nurses and offered guidance on engaging with the social media application. All participants are informed and consent to the research and have the right to withdraw at any time. Upon survey completion, participants received messages of gratitude in digital form for their contribution to the research endeavour. The study bolstered its credibility by incorporating a robust sample size of 1,300 eligible registered nurses from the hospital, thereby mitigating potential non-response biases.

### Measurements

#### Utrecht work engagement scale

The Chinese version of Schaufeli and Bakker’s (13) Utrecht Work Engagement Scale (UWES) was employed for evaluating work engagement (14). It comprises three subscale dimensions—vigor, dedication, and absorption—across sixteen items. Each item is scored on a scale ranging from 0 to 4. A Cronbach’s alpha coefficient of 0.90 was obtained. In our study, the Cronbach’s alpha for the UWES was 0.866.

#### Self-compassion scale

The Self-Compassion Scale (SC), originally developed by Neff (15), was employed in its Chinese version to assess self-compassion. Chen et al. (11) reported a retest reliability of 0.89 and a Cronbach’s alpha of 0.84. The scale consists of 26 items across six categories: self-judgment, self-kindness, isolation, mindfulness, and over-identification. Participants rated each item on a 5-point Likert scale. Higher scores indicate lower levels of negative emotions and higher levels of self-compassion. In our study, the Cronbach’s alpha for the SC was 0.875.

#### Depression-anxiety-stress scale

To measure unpleasant feelings, the Depression, Anxiety, and Stress Scale – 21 Items (DASS-21) (16) was utilized. Each dimension comprises seven questions, scored from 0 to 3, with higher scores indicating stronger negative emotions. The Chinese version of the DASS-21 has demonstrated reliability and validity (17, 18). Li et al. (19) reported a test–retest reliability of 0.751 and a Cronbach’s alpha of 0.912. In our investigation, the questionnaire’s Cronbach’s alpha was 0.916.

#### Demographic information

Demographic variables, as outlined by Yang et al. (20), included gender, age, education, marital status, years of work experience, sleep quality, and physical exercise.

#### Statistical analysis

Descriptive statistics, including means and standard deviations, were computed for participants’ demographic details and variable levels. Pearson’s correlation analysis examined relationships between variables. Structural equation modeling (SEM) assessed the mediation model, with self-compassion as the independent variable, UWES as the dependent variable, the DASS-21 as the mediating factor, and working years as the regulatory variable. The validity assessment relied on a suite of fit indices, including the chi-square statistic adjusted for degrees of freedom, the Comparative Fit Index (CFI), the Goodness of Fit Index (GFI), Adjusted Goodness of Fit Index (AGFI), the Root Mean Square Error of Approximation (RMSEA), and the Standardized Root Mean Square Residual (SRMR). Acceptable model fit is suggested by SRMR values under 0.07, RMSEA values under 0.08, and CFI, GFI, and AGFI values exceeding 0.95 (22). Additionally, convergent validity was gauged by examining the Average Variance Extracted (AVE) and composite reliability metrics, adhering to the benchmark where AVE exceeds 0.50 and reliability surpasses 0.70. AMOS 23.0 and SPSS 25.0 were utilized for the analysis.

## Results

### Demographics

A total of 1,300 nurses participants in this study, 1,201 (92.38%) wrote a response to the questionnaire question (Table 1). These respondents were 1,151 (96.10%) being female and 47 being male. The average age of participants is 32.65 (±6.90) years. 75.40% of participants are married, 85.10% hold a bachelor’s degree, 70.50% have at least one child, and 31.50% have worked for six to ten years.

**Table 1:** Demographic characteristics of the sample used in this study.

Characteristics	Total sample (*N* = 1,569)
Female, *n* (%)	1,154 (96.10)
Age, mean (SD) [range]	32.65 (6.90) [21–55]
Education, *n* (%)	
Some college	179 (14.90)
Undergraduate	1,013 (84.35)
Postgraduate	9 (0.75)
Marital status, *n* (%)	
Unmarried	278 (23.10)
Married	905 (75.40)
Divorced	15 (1.2)
Widowed	3 (0.20)
Number of children, *n* (%)	
0	355 (29.50)
1	516 (43.00)
2	328 (27.30)
≥3	2 (0.20)
Years of work experience, *n* (%)	
≤5	326 (27.20)
6–10	378 (31.50)
11–15	292 (24.30)
≥16	205 (17.00)
Sleep quality, *n* (%)	
Excellent	20 (1.70)
Good	206 (17.20)
Fair	655 (54.50)
Poor	262 (21.80)
Very poor	58 (4.80)
Physical exercise, *n* (%)	
Usually	80 (6.70)
Sometimes	207 (17.20)
Occasionally	562 (46.80)
Never	352 (29.30)
Participate in emotion management training, *n* (%)	
Usually	7 (0.60)
Sometimes	114 (9.50)
Occasionally	321 (6.70)
Never	756 (63.20)
Attend nurse self-compassion training, *n* (%)	
Usually	18 (1.50)
Sometimes	158 (13.20)
Occasionally	445 (37.10)
Never	580 (48.30)
WE, mean (SD) [range]	53.28 (5.66) [16–80]
SC, mean (SD) [range]	73.09 (6.87) [26–130]
DASS, mean (SD) [range]	32.44 (7.30) [21–84]

Psychological Flex Work Engagement Score, WE; Self-compassion, SC; Depression, Anxiety, and Stress, DASS.

### Measurement model

The effectiveness of the proposed model was assessed through confirmatory factor analysis involving all variables. Due to low factor loadings (below 0.6), two items were excluded from the Self-Compassion (SC) scale, consistent with prior research methodologies (21). Following these adjustments, the model demonstrated satisfactory fit (22).

Moreover, Tables 2, 3 present discriminant and convergent validity results, respectively (23, 24). The Average Variance Extracted (AVE) values ranged from 0.523 to 0.759, and Composite Reliability (CR) values ranged from 0.654 to 0.904, meeting the criteria for convergent validity (Table 2) (24). Table 3 exhibits the correlation matrix for the variables under investigation.

**Table 2:** Construct validity.

Construct	Dimension number	Estimate	AVE	CR
SC	Over-identified	0.690	0.589	0.654
	Isolation	0.844		
	Common Humanity	−0.365		
	Self-Judgment	0.796		
DASS	Stress	0.879		
	Anxiety	0.865	0.759	0.904
	Depression	0.870		
WE	Vigour	0.629	0.523	0.765
	Dedication	0.800		
	Absorption	0.729		

**Table 3:** Discriminatory validity.

	SC	DASS	WE
SC			
DASS	−0.456^***^		
WE	0.119^***^	−0.308^***^	
Skewness	−0.058	0.146	−0.241
Kurtosis	−0.240	−1.170	−0.707
AVE-square root	0.699	0.871	0.723

***p < 0.001: the diagonal line represents the amount of variance variation extracted for AVE evaluation.

The kurtosis values, ranging from −1.170 to −0.240 (below an absolute value of 8), and skewness values, ranging from −0.241 to 0.146 (below an absolute value of 3), indicate a normal distribution for all variables. All relationships were found to be statistically significant (*p* < 0.01), with a notably positive correlation (*p* < 0.001) observed between work engagement and self-compassion, and a negative correlation with negative emotions.

Furthermore, discriminant validity was confirmed as the square roots of the AVE exceeded the correlations between corresponding variables in Table 2. The variance inflation factor values ranged from 1.263 to 1.433, indicating the absence of multicollinearity issues.

### Structural model

**Figure 2:**
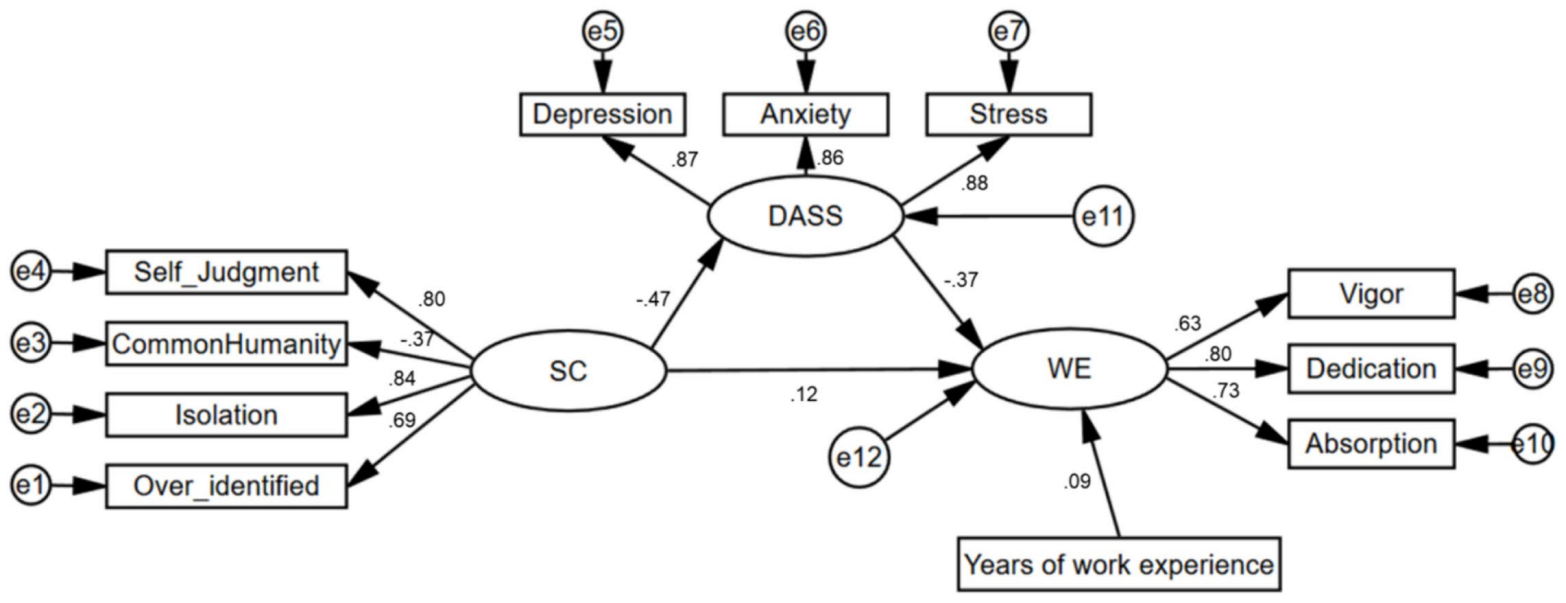
Model diagram describing that negative emotions mediate the correlation between work engagement and self-compassion.

The mediating role of DASS-21 in the relationship between SC (Social Capital) and WE (Work Engagement) was examined using Structural Equation Modeling, as depicted in Figure 2. The mediation analysis met satisfactory criteria based on the SEM fit indices: Chi-square/df = 2.724, CFI = 0.989, GFI = 0.986, AGFI = 0.972, RMSEA = 0.038, and SRMR = 0.030.

Figure 2 illustrates our findings that SC has a direct impact on DASS, which subsequently influences work engagement. There is a statistically significant correlation (β = 0.116, *p* < 0.05) between SC and WE. Additionally, there is a significant positive correlation between WE and years of work experience (β = 0.09, *p* < 0.01).

Importantly, Table 4 demonstrates that DASS serves as a mediator in the relationship between SC and WE, with a mediating effect of 0.174. The findings in Table 4 present 95% confidence intervals, indicating the significance of the indirect path coefficient.

**Table 4:** Direct and indirect effects of the final model.

Effect	Path	Estimate	*p*-value	95% Bias-corrected CI	
Direct	SC → WE	0.116	0.000	−0.036	−0.008
	DASS→WE	−0.372	0.000	−0.053	−0.033
Indirect	SC → DASS→WE	0.174	0.001	−0.067	−0.021
Total direct		0.059	0.002	−0.286	−0.068

## Discussion

In this study, we sought to explore the mediating effects of negative emotions on the relationship between self-compassion and work engagement among Chinese nurses. Our findings provide valuable insights into how these psychological factors interact and influence work engagement, which is critical for enhancing job performance and overall well-being in nursing professionals. First, our results indicate that self-compassion is positively associated with work engagement. Nurses with higher levels of self-compassion may be better equipped to manage the emotional and physical stresses of their job, leading to greater work engagement (26). Given the high-stress environment of nursing, it is not surprising that negative emotions significantly impact work engagement. Importantly, our analysis shows that negative emotions partially mediate the relationship between self-compassion and work engagement. The self-compassion group demonstrated increased work engagement attributed to a reduction in negative affect, as reported by Dreisoerner et al. (25). This suggests that while self-compassion directly enhances work engagement, it also does so indirectly by reducing negative emotions (26). Nurses who cultivate self-compassion may experience fewer negative emotions, thereby maintaining higher levels of engagement. This partial mediation indicates that interventions aimed at boosting self-compassion could be a promising strategy for not only improving nurses’ emotional well-being but also enhancing their engagement at work.

With the increasing pressure on employment and intensifying competition, work engagement is gaining more attention from employers and leaders as a valuable human resource. Numerous research endeavors have shown substantial interest in the realm of WE. This encompasses the examination of authentic leadership (AL) and its impact on work engagement, as well as the intricate relationships between task performance and organizational outcomes (27, 28). Currently, there is a limited body of research exploring the correlation between Self-compassion and Work Engagement among Chinese nurses. To address this gap, this research investigated the impact of Self-Compassion, Depression, Anxiety, and Stress on the Work Engagement of Chinese nurses through a comprehensive survey. The findings indicated a direct and adverse impact of Self-Compassion on Work Engagement among Chinese nurses. On the basis of Acceptance and Commitment Therapy (ACT) (29), self-compassion is a vital component of mental health, cultivating self-compassion can lead to improvements in various domains of psychological well-being, including reduced anxiety and depression, increased resilience, and enhanced quality of life. Self-Compassion was associated with higher levels of work engagement. Moreover, a longitudinal increase in psychological flexibility corresponded to a subsequent rise in work engagement levels (26).

This research revealed that DASS play intermediary roles in Self-Compassion and work engagement in Chinese nurses. The findings align with earlier research, corroborating the established conclusions. Mental health serves as a mediator between self-compassion and work engagement in influencing the job performance of ICU nurses (26). Cultivating self-compassion could serve as an effective strategy for safeguarding the mental well-being of employees in nations where mental health issues are inversely linked to work engagement and intrinsic motivation (30). The findings validated that physicians who practiced self-compassion demonstrated heightened levels of positive work engagement, reduced feelings of emotional, physical, and cognitive exhaustion resulting from work demands, and reported greater satisfaction with their professional lives compared to their counterparts who displayed lower levels of self-compassion amidst uncertain and challenging circumstances (7). The correlations between emotional disorders and work engagement were found to be consistently significant. Moreover, emotional disorders were identified as partial mediators in the links between conflict management styles and work engagement (31). The study conducted by Nordin et al. (32) discovered a positive relationship between psychological capital and work engagement. There was a notable correlation observed between emotional disorders and work engagement, emotional disorders were found to play a partial mediating role in the connections between conflict management styles and work engagement (31). According to Perepelkin and Wilson (33), a distinct perspective emerged indicating that burnout serves as a complete mediator in the relationship between anxiety and employee engagement. This implies that anxiety by itself does not suffice to diminish employee engagement. This outcome aligned with conclusions drawn from various other research investigations. These results imply that cultivating a strong sense of self-compassion and mental health could enhance one’s level of work engagement. Furthermore, it was found in this study that years of work experience play a moderating role in work engagement, which is consistent with previous research. According to Wang et al. (34), the proportion of nurses with five or more years of experience, known as skill mix, had a significant impact on engagement. It was found that participants with less than five years of nursing experience contributed significantly to the variation in work engagement (35). In comparison to those employed for fewer than five years, individuals with five to nine years of experience experienced a notably higher level of burnout, which was negatively associated with health (36).

## Limitations

There are several restrictions on the current investigation. First, our investigation, while built upon the findings of existing research, was inherently limited by its cross-sectional approach, which precluded the determination of causal linkages between the variables in question. Second, the application of a non-probability sampling technique, specifically convenience sampling, has confined the scope of our results’ generalizability. Third, our research indicates that years of work experience have a moderating effect on job engagement and positively influence it in the structural equation model in the current investigation. However, we did not conduct a stratified analysis based on years of work experience to explore the impact of varying tenure levels on job engagement. Future research in this area is warranted.

## Conclusions and implications to practice

Based on our analysis of a substantial sample of Chinese nurses, this study illuminates the relationship between negative emotions, self-compassion, and work engagement. It also offers insights into intervention strategies based on Acceptance and Commitment Therapy (ACT) aimed at enhancing nurses’ work engagement.

Our research reveals a significant positive correlation between work engagement and self-compassion. Additionally, years of work experience exert influence on work engagement, while negative emotions serve as a mediator between self-compassion and work engagement.

Our findings suggest that high levels of self-compassion, a supportive work environment, and emotional regulation can enhance work engagement and increase productivity. Therefore, nursing managers should prioritize the physical and emotional well-being of their staff. This can be achieved by implementing policies that safeguard their health and cultivate a positive work environment, ultimately boosting employee engagement and productivity.

The insights from this study provide hospital administrators and nurses with a solid framework for enhancing work engagement among their colleagues.

## Data Availability

The raw data supporting the conclusions of this article will be made available by the authors, without undue reservation.
